# A universal methodology for reliable predicting the non-steroidal anti-inflammatory drug solubility in supercritical carbon dioxide

**DOI:** 10.1038/s41598-022-04942-4

**Published:** 2022-01-20

**Authors:** Tahereh Rezaei, Vesal Nazarpour, Nahal Shahini, Soufia Bahmani, Amir Shahkar, Mohammadreza Abdihaji, Sina Ahmadi, Farzad Tat Shahdost

**Affiliations:** 1 Neuroscience Research Center, Shiraz University of Medical Sciences, Shiraz, Iran; 2 Department of Biomedical Engineering, Mashhad Branch, Islamic Azad University, Mashhad, Iran; 3 Department of Computer Engineering, Amirkabir University of Technology, Tehran, 15875-4413 Iran; 4 Department of Transportation Engineering, Karadeniz Technical University, 61080 Trabzon, Turkey; 5 Department of Biology, The Center for Genomics and Bioinformatics, Indiana University, Bloomington, IN USA; 6 Department of Computer Engineering, West Tehran Branch, Islamic Azad University, Tehran, Iran; 7 Department of Electrical Engineering, Garmsar Branch, Islamic Azad University, Semnan, Iran

**Keywords:** Pharmaceutics, Chemical engineering, Medicinal chemistry

## Abstract

Understanding the drug solubility behavior is likely the first essential requirement for designing the supercritical technology for pharmaceutical processing. Therefore, this study utilizes different machine learning scenarios to simulate the solubility of twelve non-steroidal anti-inflammatory drugs (NSAIDs) in the supercritical carbon dioxide (SCCO_2_). The considered NSAIDs are Fenoprofen, Flurbiprofen, Ibuprofen, Ketoprofen, Loxoprofen, Nabumetone, Naproxen, Nimesulide, Phenylbutazone, Piroxicam, Salicylamide, and Tolmetin. Physical characteristics of the drugs (molecular weight and melting temperature), operating conditions (pressure and temperature), and solvent property (SCCO_2_ density) are effectively used to estimate the drug solubility. Monitoring and comparing the prediction accuracy of twelve intelligent paradigms from three categories (artificial neural networks, support vector regression, and hybrid neuro-fuzzy) approves that adaptive neuro-fuzzy inference is the best tool for the considered task. The hybrid optimization strategy adjusts the cluster radius of the subtractive clustering membership function to 0.6111. This model estimates 254 laboratory-measured solubility data with the AAPRE = 3.13%, MSE = 2.58 × 10^–9^, and R^2^ = 0.99919. The leverage technique confirms that outliers may poison less than four percent of the experimental data. In addition, the proposed hybrid paradigm is more reliable than the equations of state and available correlations in the literature. Experimental measurements, model predictions, and relevancy analyses justified that the drug solubility in SCCO_2_ increases by increasing temperature and pressure. The results show that Ibuprofen and Naproxen are the most soluble and insoluble drugs in SCCO_2_, respectively.

## Introduction

Separation scenarios, including fluidization^1^, liquid–liquid extraction^2^, adsorption^3,4^, crystallization^5^, membrane^6,7^, and microfluid absorption^8^, are continuously engaged in different industrial processes. Moreover, the processes operated with the supercritical fluids have a wide range of applications in diverse fields, including extraction^9^, reaction^10^, food industry^11^, nanoparticle decoration^12^, nanosheet fabrication^13^, tissue engineering^14^, and pharmaceutical processing^15^. Water^16^, propane^17^, and carbon dioxide (CO_2_)^18^ are among materials potentially used as the supercritical medium. The unique characteristics, such as mild critical temperature (31.1 °C) and pressure (73.8 bar)^19^, provide carbon dioxide with diverse applications as a supercritical solvent^20^. Furthermore, carbon dioxide in the supercritical state is a low-cost and low viscous solvent with high diffusivity and solvating ability^21^.

Application and interest in using the supercritical CO_2_ (SCCO_2_) for pharmaceutical processing have been sharply increased recently^15,22–28^. Understanding the drug solubility in SCCO_2_ is the central information for designing the supercritical-based pharmaceutical technology^29^. The size^26^, shape^26^, surface structure^22^, morphology^22^, and crystallization process^26^ of synthesized solid drugs are determined by their solubility in the supercritical fluid. In addition, the economic success of the supercritical technology highly depends on reliable insight about the solid (drug) solubility in supercritical solvents^23^.

Therefore, some researchers focused on laboratory measurements of solid drug solubility in supercritical CO_2_^15,22–28^. However, experimental determination of pharmaceutical solubility in SCCO_2_ is complex, expensive, and time-consuming^23,30^. In addition, it is not possible to measure equilibrium solubility in all ranges of desired operating conditions^26,30^.

Hence, several empirical^31,32^ and thermodynamic-based^23,33^ correlations have been proposed to calculate the solid drug solubility in the CO_2_ at the supercritical state. Traditionally equations of state are the most utilized thermodynamic-based correlations for predicting the phase equilibria of drugs/SCCO_2_^34–36^. Unfortunately, these thermodynamic-based methods have at least one temperature-dependent interaction parameter that must be adjusted appropriately^23^. Surprisingly, there is no general thermodynamic-based method for effectively monitoring the solubility of several solid drugs in SCCO_2_^23^. Furthermore, it is claimed that equations of state often provide high levels of uncertainty^34^ and sometimes wholly fail^35^. On the other hand, available empirical correlations have usually been developed for estimating the solubility of a specific solid drug in supercritical CO_2_, and it is impossible to find which correlation is better to use^22^.

The non-steroidal anti-inflammatory drugs (NSAID) are often prescribed to reduce pain/fever/inflammation and prevent blood clots^26^. The current research intends to propose a universal intelligent model to predict the solubility of twelve NSAIDs (Fenoprofen, Flurbiprofen, Ibuprofen, Ketoprofen, Loxoprofen, Nabumetone, Naproxen, Nimesulide, Phenylbutazone, Piroxicam, Salicylamide, and Tolmetin) in SCCO_2_. For doing so, 2150 intelligent paradigms from three different categories (i.e., artificial neural networks, hybrid neuro-fuzzy, and support vector regression) have been constructed, and their accuracy monitored. The ANFIS model with the subtractive clustering membership function and cluster radius of 0.6111 presents the most reliable prediction results. This straightforward model can accurately predict the solubility of 12 NSAIDs in supercritical CO_2_ in wide ranges of operating pressures and temperatures. To the best of our knowledge, it is the most generalized approach developed for phase equilibria modeling of NSAIDs/SCCO_2_ up to now.

## Material and methods

The collected drug solubility data, their sources, and ranges of experimental measurements have been reported in this section. Furthermore, the current section has concisely introduced the applied machine learning methods.

### Experimental data for anti-inflammatory drug solubility in SCCO_2_

Development, as well as validation stages of all machine learning techniques, require an experimentally measured databank about the given problems. Therefore, in the current research, the information of 254 experiments related to the anti-inflammatory drug solubility in supercritical CO_2_ has been gathered from eight trusted literature^15,22–28^. A complete description of these experiments, including their range of operating pressures and temperatures, the observed solubility levels, and numbers of available data for all anti-inflammatory drug/SCCO_2_ systems, have been introduced in Table 1. It is also necessary to highlight that subscript 1 and 2 are associated with the anti-inflammatory drug and supercritical carbon dioxide, respectively.

**Table 1:** Available laboratory measurements for solubility of anti-inflammatory drugs in supercritical CO_2_.

CO_2_ (1) + drug (2)	Temperature range (K)	Pressure range (MPa)	Solubility range (mole fraction)	No. of data
Fenoprofen^22^	308.00–338.00	12.00–40.00	0.000020–0.004200	32
Flurbiprofen^23^	303.00–323.00	8.90–24.50	0.000017–0.000197	27
Ibuprofen^24^	313.15–313.15	12.12–23.1	0.002100–0.007700	9
Ketoprofen^25^	312.50–331.50	10.00–22.00	0.000013–0.000155	10
Loxoprofen^26^	308.00–338.00	12.00–40.00	0.000014–0.001280	32
Nabumetone^27^	308.20–328.20	10.00–22.00	0.000039–0.002680	21
Naproxen^24^	313.15–313.15	12.11–27.98	0.000010–0.000042	9
Nimesulide^25^	313.10–333.10	13.00–22.00	0.000019–0.000099	8
Phenylbutazone^27^	308.20–328.20	10.00–22.00	0.000020–0.002650	21
Piroxicam^25,28^	308.15–338.15	13.00–40.00	0.000012–0.000512	37
Salicylamide^27^	308.20–328.20	10.10–22.00	0.000028–0.000210	21
Tolmetin^15^	308.00–338.00	12.00–40.00	0.000019–0.002590	32

Since the solubility of all anti-inflammatory drugs in supercritical CO_2_ is planned to be estimated by a single model, it is necessary to include the drugs’ inherent characteristics in the modeling stage, too. Table 2 shows the molecular weight and melting temperature of the considered anti-inflammatory drugs. It is better to note that each anti-inflammatory drug has its unique values for these properties. Therefore, the molecular weight and melting temperature can be incorporated in the model’s entry to differentiate among different anti-inflammatory drugs.

**Table 2:** Physical properties of the considered anti-inflammatory drugs.

Anti-inflammatory drug	Molecular weight (g/mole)	Melting temperature (K)
Fenoprofen	244.27	386.15
Flurbiprofen	206.00	347.65
Ibuprofen	254.29	367.65
Ketoprofen	246.10	383.00
Loxoprofen	228.30	353.15
Nabumetone	230.00	430.65
Naproxen	308.31	421.65
Nimesulide	308.30	378.58
Phenylbutazone	331.30	469.15
Piroxicam	137.10	413.58
Salicylamide	257.29	429.00
Tolmetin	244.27	386.15

Although it is possible to extract some features from the experimental database^37^ and utilize them as model’s entry, the current research aims to relate anti-inflammatory drug solubility in SCCO_2_ ($y_{2}$) to the molecular weight ($Mw_{2}$), melting temperature ($Tm_{2}$), operating pressure (P), temperature (T), and SCCO_2_ density ($\rho_{1}$). The mathematical statement of this expression is shown by Eq. (1).

1 $$y_{2} \, = \,f_{ML} \,\left( {Mw_{2} ,\,Tm_{2} ,\,T,\,P,\,\rho_{1} } \right)$$

Three trustful relevancy analysis approaches, namely Spearman, Pearson, and Kendal, have been utilized to check whether the selected independent variables are appropriate features for the model development^38^. These techniques show the relevancy level between a pair of dependent-independent variables by a coefficient in the range of minus one to plus one^39^. The negative coefficients indicate indirect dependency, positive ones show a direct relationship, and zero coefficient value is associated with no relevancy^39^.

**Figure 1:**
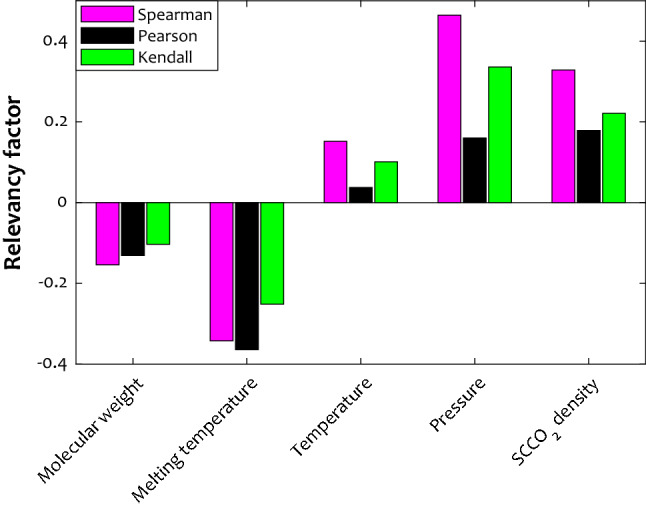
The value of Spearman, Pearson, and Kendall factors for relevancy between drug solubility and the corresponding influential variables.

Figure 1 presents the observed coefficients of Spearman, Pearson, and Kendall techniques for interrelations of the anti-inflammatory drug solubility in SCCO_2_ with the selected independent variables. This analysis approves that increasing the molecular weight and melting temperature of anti-inflammatory drugs reduces their dissolution in the supercritical CO_2_. On the other hand, raising pressure, temperature, and solvent density enhance drug solubility in the SCCO_2_. Furthermore, molecular weight and pressure have the weakest indirect and strongest direct influences on the drug solubility in the SCCO_2_, respectively. The performed relevancy analysis results can be considered a justification for the appropriate selection of the independent variables.

### Computational methodologies

Machine learning methods have been extensively engaged in approximation^40,41^, interpretation^42^, action recognition^43^, and classification^44,45^ porpuses. This study focuses on five artificial neural networks (ANN), four hybrid neuro-fuzzy types, and three kinds of support vector regression (SVR) to simulate anti-inflammatory drug solubility in supercritical CO_2_. The considered ANN models are multilayer perceptron neural network (MLPNN)^46,47^, cascade feedforward neural network (CFFNN)^48^, recurrent neural network (RNN)^49,50^, general regression neural network (GRNN)^48^, and radial basis function neural networks (RBFNN)^51^. The efficiency of the support vector regression with the linear kernel (LSSVR-L)^52^, polynomial kernel (LSSVR-P)^52^, and Gaussian kernel (LSSVR-G)^53^ are also evaluated over the considered purpose. The neuro-fuzzy models with the subtractive clustering membership function trained by the hybrid (ANFIS2-H) and backpropagation (ANFIS2-BP) algorithms have also been applied in the current study^54^. The last intelligent tools used in the present research are the neuro-fuzzy models with the C-means clustering membership function trained by hybrid (ANFIS3-H) and backpropagation (ANFIS3-BP) optimization strategies^55^.

It should be mentioned that these paradigms can be viewed as advanced regression-based tools. Therefore, they have all limitations of the conventional regression-based methods. Indeed, the developed intelligent schemes are only valid for the ranges of experimental data reported in Table 1. Utilizing these models for extrapolation purposes is not suggested.

## Results and discussions

The focus of the present section is devoted to constructing different numbers of the considered intelligent paradigms through the trial-and-error tactic and determining models with the lowest deviation from experimental measurements. Then the model with the highest accuracy is found applying the ranking analysis. After this, several visual inspections have been directed to evaluate the selected model efficiency for estimating anti-inflammatory drugs’ solubility in supercritical CO_2_. The ability of the fabricated intelligent model to recall the physical-based behavior of the anti-inflammatory drug in the supercritical fluid (variation of drug solubility by the operating conditions) has also been inspected in the present section.

### Smart models’ construction

The present research employs five types of artificial neural networks (MLPNN, CFNN, RNN, GRNN, and RBFNN), three support vector regression kinds (LSSVR-L, LSSVR-P, and LSSVR-G), and four hybrid neuro-fuzzy approaches (ANFIS2-H, ANFIS2-BP, ANFIS3-H, and ANFIS3-BP) for simulating the anti-inflammatory drugs’ solubility in the supercritical CO_2_. All these intelligent tools have their own unique features required to be appropriately determined. Table 3 expresses both fixed and tunable elements of the applied machine learning methodologies in the present research. This table also indicates the range of the tunable features of the intelligent paradigms during the trial-and-error process. The last column of Table 3 shows the numbers of the constructed models for all individual smart categories. Cumulatively, 2150 intelligent estimators have been fabricated during the development stage.

**Table 3:** Complete information about 2150 constructed computational techniques by the trial-and-error procedure.

AI model	Fixed parameters	Deciding parameters	No. of models
MLPNN	Two neuronic layers Levenberg–Marquardt optimization scenario Tangent and logarithm sigmoid activation function	1–10 Hidden neurons Weights and biases	300
CFFNN	Two neuronic layers Levenberg–Marquardt optimization scenario Tangent and logarithm sigmoid activation functions	1–9 Hidden neurons Weights and biases	180
RNN	Two neuronic layers Scaled Conjugate Gradient optimization scenario Tangent and logarithm sigmoid activation functions	1–6 Hidden neurons Weights and biases	120
GRNN	Two neuronic layers Gaussian and linear activation functions	1 × 10^–6^ < Spread factor < 10 Weights and biases	200
RBFNN	Two neuronic layers Gaussian and linear activation functions	1–10 Hidden neurons 1 × 10^–6^ < Spread factor < 10 Weights and biases	250
ANFIS2-H	Subtractive clustering membership function Hybrid optimization scenario	0.5 < Radius of cluster < 1 Membership function parameters	200
ANFIS2-BP	Subtractive clustering membership function Backpropagation optimization scenario	0.5 < Radius of cluster < 1 Membership function parameters	200
ANFIS3-H	C-means clustering membership function Hybrid optimization scenario	2–11 Cluster Membership function parameters	200
ANFIS3-BP	C-means clustering membership function Backpropagation optimization scenario	2–11 cluster Membership function parameters	200
LSSVR-L	Linear kernel function	Weights and biases Linear kernel parameter	100
LSSVR-P	Polynomial kernel function	Weights and biases Polynomial kernel parameters	100
LSSVR-G	Gaussian kernel function	Weights and biases Gaussian kernel parameters	100

#### Training process

The actions followed to adjust hyperparameters of machine learning methods is known as the training process^56^. This process utilizes historical data of a given phenomenon and an optimization algorithm to perform this duty. The literature has already compared the accuracy and computation time of some well-known training algorithms engaged in the training stage of machine learning methods^56^. The training stage begins with randomly generated hyperparameters. The estimated targets have been obtained by entering independent variables into an intelligent estimator. The deviation between the calculated and actual values of the dependent variable is considered an objective function of the optimization algorithm. Indeed, the optimization algorithm continuously updates the hyperparameters of the machine learning method to minimize the objective function or at least reduce it as much as possible. The training stage finishes when the maximum number of iterations is reached or the objective function converges to the prespecified value^57^.

A trained machine learning method is then possible to employ for estimating the target variable in unknown situations. All trained intelligent tools only require the independent variables to do their duty.

It can be understood from Table 3 that the radial basis function and general regression neural networks, and support vector regression benefit from the Gaussian function^58^. Indeed, the first two models have the Gaussian-shape activation function, but the latest uses the Gaussian as the kernel function.

### Smart models’ selection

In order to find the best structure of each smart method, it is necessary to quantize the prediction errors of the engineered models using appropriate statistical criteria. Those models provided the lowest prediction errors finally selected as the best ones. In this way, it is also possible to determine the most appropriate structural features. Table 4 presents the final twelve smart paradigms (one model per category) with the slightest prediction errors. This table also displays the prediction errors of these selected models in terms of six uncertainty criteria (AAPRE%, MAE, RAE%, RRSE%, MSE, and R^2^). The calculated uncertainties have been separately reported for the training and testing categories. Equations (2) to (7) express that only laboratory-measured ($y_{{_{2} }}^{\exp }$) and calculated ($y_{{_{2} }}^{cal}$) drug solubility, numbers of data (N), and the average value of solubilities ($\overline{{y_{{_{2} }}^{\exp } }}$) are needed to quantize these accuracy criteria^38,59^.

2 $$R^{2} = \, - \sum\limits_{r = 1}^{N} {\left( {y_{{_{2} }}^{\exp } - y_{{_{2} }}^{cal} } \right)_{r}^{2} /\sum\limits_{r = 1}^{N} {\left( {y_{{_{2} }}^{\exp } - \overline{{y_{{_{2} }}^{\exp } }} } \right)_{r}^{2} } } \, + \,1$$

3 $$RAE\% = 100\, \times \,\,\left[ {\sum\limits_{r = 1}^{N} {\left| {y_{{_{2} }}^{\exp } - y_{{_{2} }}^{cal} } \right|_{r} /\sum\limits_{r = 1}^{N} {\left| {y_{{_{2} }}^{\exp } - \overline{{y_{{_{2} }}^{\exp } }} } \right|_{r} } } } \right]$$

4 $$MSE = \sum\limits_{r = 1}^{N} {\left( {y_{{_{2} }}^{\exp } - y_{{_{2} }}^{cal} } \right)_{r}^{2} } /N$$

5 $$MAE = \,\,\sum\limits_{r = 1}^{N} {\left| {y_{{_{2} }}^{\exp } - y_{{_{2} }}^{cal} } \right|}_{r} /N$$

6 $$AAPRE\% = \left( {100/N} \right)\, \times \sum\limits_{r = 1}^{N} {\left( {\left| {y_{{_{2} }}^{\exp } - y_{{_{2} }}^{cal} } \right|/y_{{_{2} }}^{\exp } } \right)_{r} }$$

7 $$RRSE\% = 100\, \times \,\,\sqrt {\sum\limits_{r = 1}^{N} {\left( {y_{{_{2} }}^{\exp } - y_{{_{2} }}^{cal} } \right)_{r}^{2} /\sum\limits_{r = 1}^{N} {\left( {y_{{_{2} }}^{\exp } - \overline{{y_{{_{2} }}^{\exp } }} } \right)_{r}^{2} } } } .$$

**Table 4:** The best-selected property for the employed intelligent models and their related prediction accuracy.

Model	Best feature	Group	AAPRE%	MAE	RAE%	RRSE%	MSE	R^2^
MLPNN	Nine hidden neurons	Training stage	9.03	6.66 × 10^–5^	8.25	11.9	2.49 × 10^–8^	0.99574
		Testing stage	18.34	1.10 × 10^–4^	21.00	27.8	3.19 × 10^–8^	0.96206
CFFNN	Seven hidden neurons	Training stage	13.31	6.07 × 10^–5^	8.01	9.4	1.40 × 10^–8^	0.99574
		Testing stage	17.68	1.02 × 10^–4^	12.91	17.0	4.20 × 10^–8^	0.98940
RNN	Five hidden neurons	Training stage	35.91	1.59 × 10^–4^	24.17	33.5	1.36 × 10^–7^	0.94773
		Testing stage	35.63	3.24 × 10^–4^	25.76	32.7	3.56 × 10^–7^	0.94759
GRNN	216 Hidden neurons Spread factor = 0.00013	Training stage	0.00	0.00	0.00	0.0	0.00	1.00000
		Testing stage	26.05	9.45 × 10^–5^	26.94	33.0	2.17 × 10^–8^	0.97892
RBFNN	Ten hidden neurons Spread factor = 0.4167	Training stage	84.99	4.24 × 10^–4^	56.50	77.6	8.41 × 10^–7^	0.66882
		Testing stage	84.12	4.87 × 10^–4^	57.97	77.2	1.51 × 10^–6^	0.74943
ANFIS2-H	Cluster radius = 0.6111	Training stage	3.04	1.99 × 10^–5^	2.48	4.2	2.87 × 10^–9^	0.99915
		Testing stage	3.69	1.49 × 10^–5^	2.82	2.9	9.6 × 10^–10^	0.99963
ANFIS2-BP	Cluster radius = 0.5556	Training stage	10.43	8.66 × 10^–5^	10.92	14.8	3.50 × 10^–8^	0.98975
		Testing stage	47.79	1.73 × 10^–4^	29.44	24.6	8.21 × 10^–8^	0.96944
ANFIS3-H	Eight clusters	Training stage	10.50	5.88 × 10^–5^	8.59	11.1	1.47 × 10^–8^	0.99390
		Testing stage	13.34	1.09 × 10^–4^	8.98	10.4	3.80 × 10^–8^	0.99553
ANFIS3-BP	Nine clusters	Training stage	25.16	2.15 × 10^–4^	29.13	45.0	2.81 × 10^–7^	0.89418
		Testing stage	47.29	1.96 × 10^–4^	21.51	31.7	2.60 × 10^–7^	0.95163
LSSVR-L	γ = 2.247	Training stage	121.19	7.16 × 10^–4^	101.00	158.2	3.63 × 10^–6^	0.14052
		Testing stage	78.95	7.66 × 10^–4^	75.69	103.4	2.28 × 10^–6^	0.64925
LSSVR-P	γ = 4.58 × 10^3^, σ^2^ = [0.5004 3]	Training stage	41.35	5.26 × 10^–4^	67.53	186.0	5.88 × 10^–6^	0.88485
		Testing stage	59.27	2.73 × 10^–4^	40.91	55.5	2.51 × 10^–7^	0.85235
LSSVR-G	γ = 436.9, σ^2^ = 0.7322	Training stage	14.16	8.45 × 10^–5^	11.93	18.4	4.59 × 10^–8^	0.99148
		Testing stage	41.23	2.43 × 10^–4^	22.65	35.2	3.42 × 10^–7^	0.96612

### Ranking analysis for finding the highest accurate smart model

The previous two sections applied a coupling technique based on the trial-and-error process and accuracy tracking to find the best topology of each smart machine. Indeed, twelve models with the highest accuracy have been extracted from 2150 fabricated approaches.

**Figure 2:**
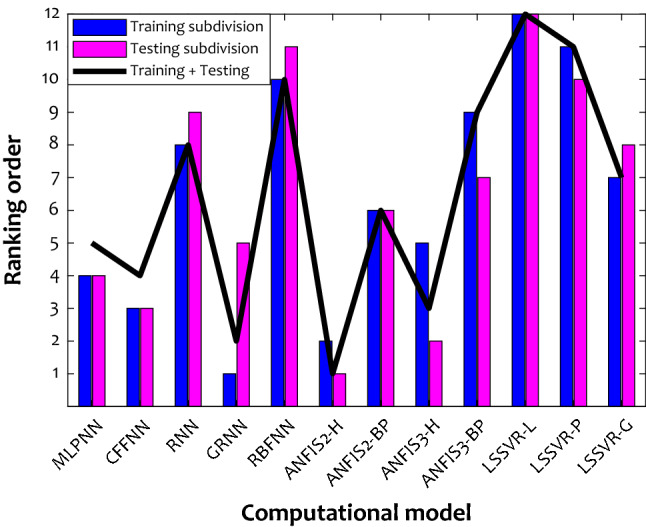
Ranking orders of the selected intelligent strategies in the learning and testing steps as well as over the whole of the datasets (testing + training).

The ranking technique is directed to find the most accurate estimator among these twelve smart methods. The outcome of performing the ranking technique on the reported results in Table 4 has been plotted in Fig. 2. Indeed, AARPE%, MAE, RAE%, RRSE%, and R^2^ with the same weight have been utilized for conducting this ranking analysis. The GRNN and ANFIS2-H are the first ranked during the training and testing stages, respectively. On the other hand, the worst model is the LSSVR-L, with the twelve ranking places for training and testing. The GRNN fails to extend its excellent ability in the training step to the testing phase (it places at the fifth ranking). This finding may indicate the overfitting of the GRNN with the 216 hidden neurons and spread index of 1.3 × 10^–4^. The ANFIS2-H efficiency in the testing stage is better than its performance in the training stage (second and first rankings in the training and testing phases). Figure 2 also indicates the performance of the selected intelligent approaches for the combination of the testing and training datasets.

It can be easily realized that the hybrid neuro-fuzzy model trained by the hybrid optimization methodology (ANFIS2-H) has the highest accuracy among 2150 initially constructed models. As Tables 3 and 4 report, this hybrid neuro-fuzzy tool has the Subtractive clustering membership function, and its adjusted cluster radius is 0.6111. This optimized topology machine provides AAPRE = 3.13%, MAE = 1.92 × 10^–5^, RAE = 2.51%, RRSE = 4.06%, MSE = 2.58 × 10^–9^, and R^2^ = 0.99919 for simulating twelve anti-inflammatory drugs’ solubility in SCCO_2_.

### Performance evaluation

**Figure 3:**
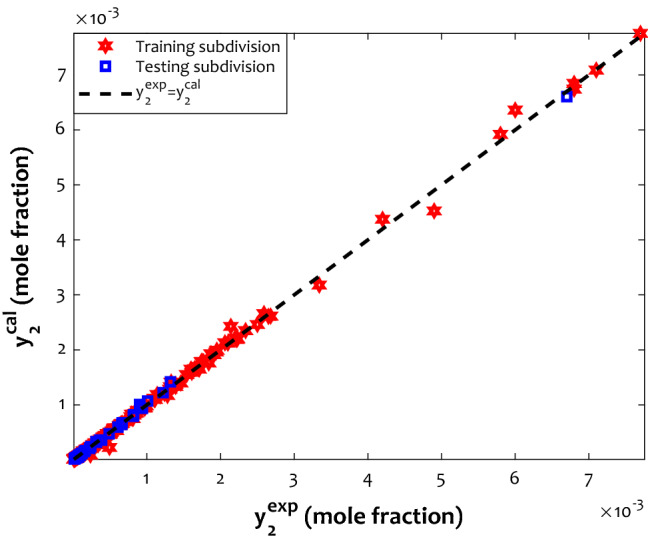
The calculated versus experimental values of the anti-inflammatory drug solubility in supercritical CO_2_.

This section concentrates on different graphical inspections to visually investigate the proposed ANFIS2-H’s performance. The cross-plot for calculated and actual drug solubilities in the SCCO_2_ have been separately depicted for the development (training) and validation (testing) stages in Fig. 3. The legend of Fig. 3 shows that the red hexagonal symbols show training subdivision, while the blue squared symbols are associated with the testing phase. Revisit the reported results in Table 4 clears that the regression coefficients for the development and validation stages are 0.99915 and 0.99963, respectively. It is clear that the constructed ANFIS2-H approach accurately estimated both databases, i.e., training and testing subdivisions.

**Figure 4:**
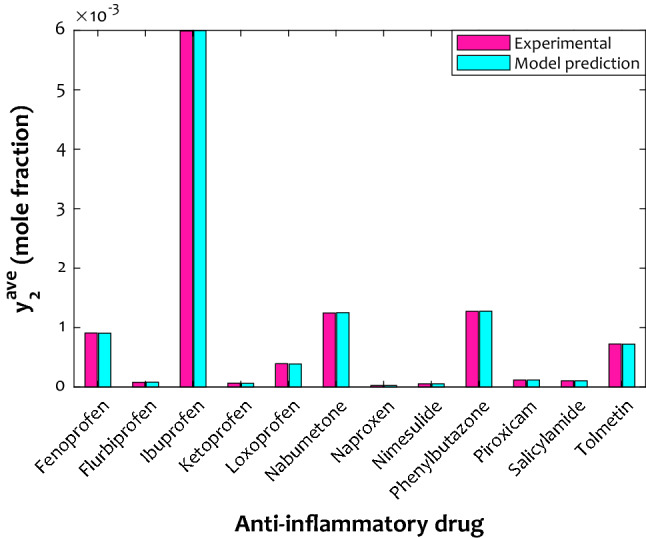
Average values of the laboratory-measured and calculated drug solubility in the considered supercritical system.

Average values of solubility of the concerned anti-inflammatory drugs in the supercritical CO_2_ for experimental measurements and ANFIS2-H predictions have been illustrated in Fig. 4. This figure can readily approve a satisfactory agreement between actual measurements and the proposed model predictions. Moreover, it can be seen that Ibuprofen and Naproxen are the most soluble and low soluble anti-inflammatory drugs in the SCCO_2_. Nabumetone and Phenylbutazone with an almost equal average solubility level are the subsequent high soluble drugs in the considered supercritical fluid.

**Figure 5:**
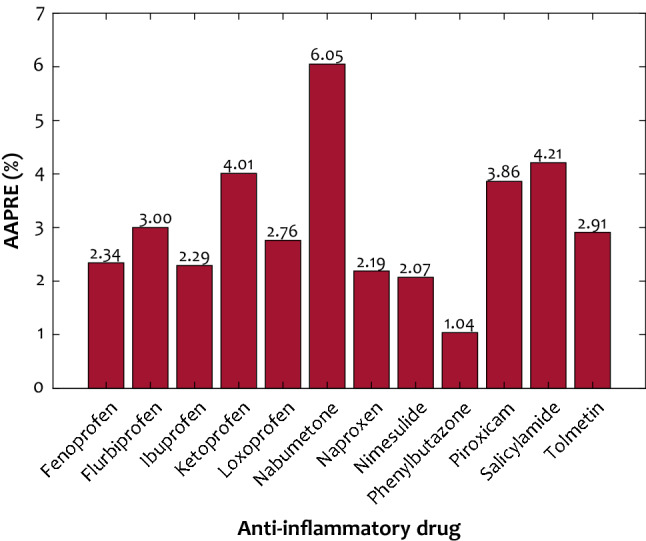
The ANFIS2-H uncertainty in terms of AAPRE for estimating the phase equilibria of all drug/SCCO_2_ systems.

The capability of the generated ANFIS2-H with the optimized topology for estimating the phase equilibria of all possible drug/SCCO_2_ systems has been depicted in Fig. 5. This figure exhibits the model’s capability in terms of AAPRE%. It can be seen that the drug/SCCO_2_ phase equilibria are simulated with the AAPRE ranges from 1.04% (Phenylbutazone) to 6.05% (Nabumetone). As mentioned earlier, an overall AAPRE of the developed ANFIS2-H for predicting 254 solubility datasets is 3.13%. It should be noted that an AAPRE of lower than 10% is an acceptable accuracy from the modeling perspective. Meanwhile, the highest observed uncertainty for predicting the Nabumetone solubility in supercritical carbon dioxide may be associated with either accompanied measurement error in experimental data or ANFIS2-H inability to estimate the Nabumetone/SCCO_2_ equilibrium accurately.

### Investigating the physical-based ability of the ANFIS2-H

The solubility of anti-inflammatory drugs in the given supercritical fluid is affected by the operating conditions, i.e., pressure and temperature. This physical-based behavior is investigated from experimental and modeling perspectives. Indeed, this section explores the ability of the designed NAFIS2-H model for correct tracing this type of behavior.

**Figure 6:**
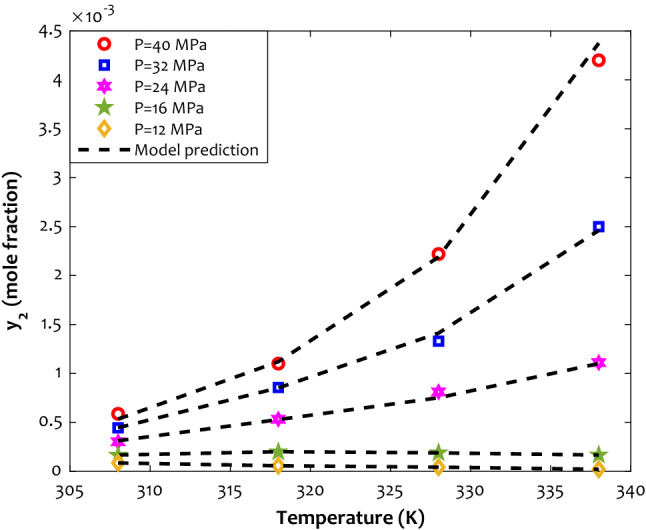
Phase behavior of the Fenoprofen/SCCO_2_ binary system in different operating conditions.

The variation of Fenoprofen solubility in the supercritical CO_2_ by the isobaric temperature alteration has been shown in Fig. 6. This figure states that the ANFIS2-H successfully understands and persuades the physical behavior of the Fenoprofen/SCCO_2_ system at different operating conditions. Moreover, this figure explains that the Fenoprofen solubility in the concerned supercritical fluid increases by increasing pressure as well as temperature. The positive effect of the temperature on the drug solubility improves by increasing the pressure. It can be claimed that the highest amount of solubility in the SCCO_2_ is achievable at the maximum allowable pressure and temperature.

It is worth noting that all other anti-inflammatory drugs also show a similar response to the alteration of the pressure/temperature. These experimental and modeling discoveries fully agree with the previously anticipated results by the relevancy analysis (“Experimental data for anti-inflammatory drug solubility in SCCO_2_” Section).

Endothermic drugs’ dissolution in the supercritical carbon dioxide may be responsible for the increasing effect of the temperature. On the other hand, increasing the pressure increases the mass driving force to transfer the drug’s molecules to the supercritical phase. Increasing the density of the supercritical fluid by increasing the pressure may be seen as another responsible for this observation.

**Figure 7:**
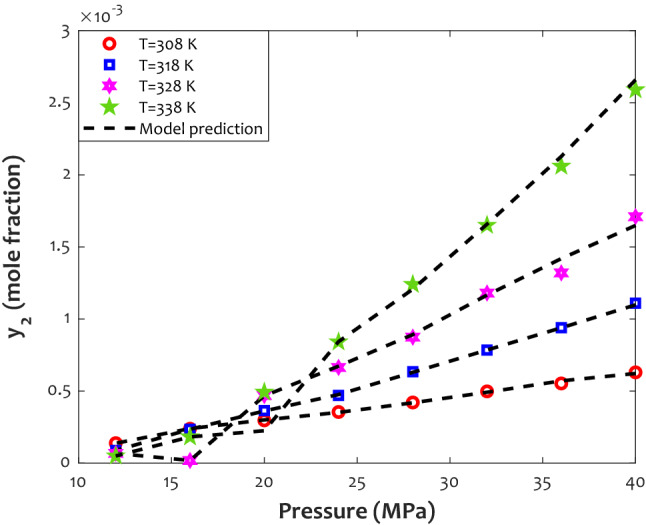
Experimental and modeling tracking of the pressure–temperature phase behavior of the Tolmetin/SCCO_2_ system.

The influence of isothermal pressure alteration on the Tolmetin dissolution in carbon dioxide in the supercritical state has been exhibited in Fig. 7. Excellent compatibility between laboratory-measured data points and ANFIS2-H predictions is observable from this figure. Like the previous analysis, the Tolmetin solubility in the SCCO_2_ continuously intensifies by raising pressure or temperature. It can also be observed that the effect of pressure on the drug solubility at high temperatures is stronger than the lower ones.

**Figure 8:**
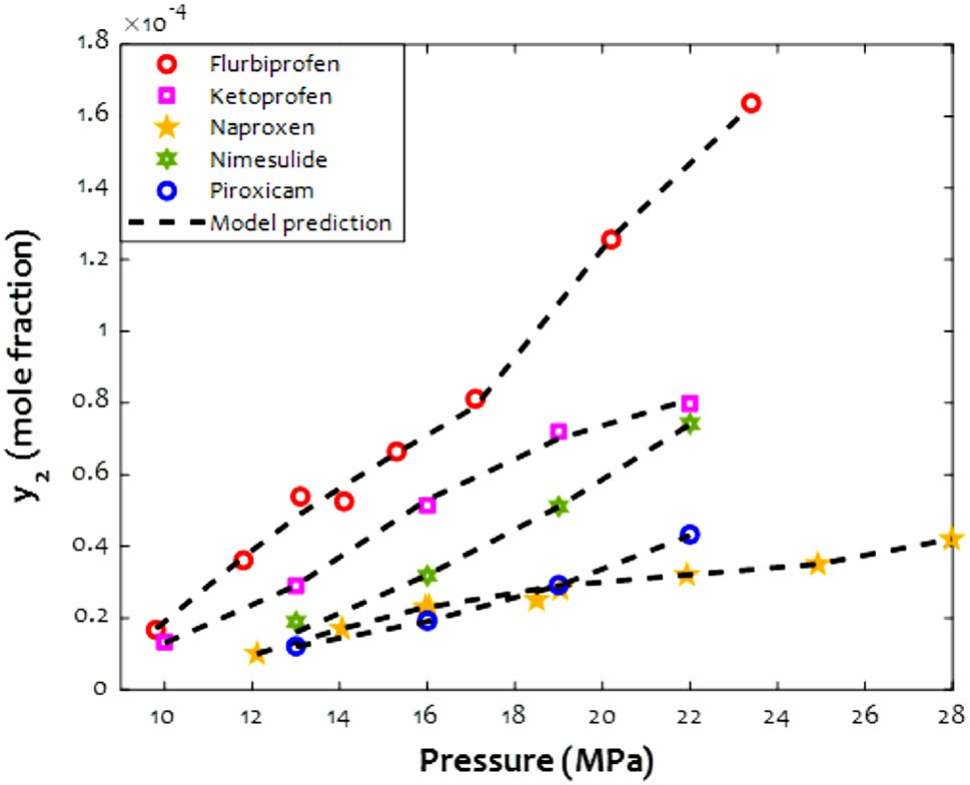
The way that anti-inflammatory drug solubility in supercritical CO_2_ changes by the pressure (T = 313.15 K).

As previously stated, the drug type also affects the magnitude of the solubility in supercritical CO_2_. The y_2_-pressure profiles of several anti-inflammatory drugs in the presence of CO_2_ in the supercritical state have been presented in Fig. 8. This figure shows outstanding compatibility between laboratory-measured information and those results calculated by the designed ANFIS2-H machine. Indeed, the proposed estimator easily distinguishes/discriminates the solubility of different anti-inflammatory drugs in the SCCO_2_. This figure easily justifies the gradual increase of the anti-inflammatory drugs’ solubility by equilibrium pressure.

### Analyzing data validity

Machine learning strategies gain their knowledge from the historical behavior of a concerning phenomenon (here, anti-inflammatory drug solubility in CO_2_ at supercritical state). Experimentations have the highest importance level to provide machine learning strategies with such insights. On the other hand, the laboratory-measured or real-field historical data is inevitably poisoned by outliers^60^. The measurement error, instrument’ wrong calibration, and environmental side effects on the experimentation are the primary sources of the outlier^52^. If the outlier information highly poisons an experimental databank used for model development, the reliability of the constructed approach is under question. Hence, the leverage tactic is suggested to inspect the validity of the experimental data^56^. This tactic plots the standard residual (SR) against the Hat index (H) to find valid as well as suspect information. Equations (8) to (11) define the formula of these variables.

8 $$RE^{ave} \,\, = \,\left( {1/N} \right)\, \times \,\sum\limits_{r = 1}^{N} {\left( {y_{{_{2} }}^{\exp } - y_{{_{2} }}^{cal} } \right)_{r} }$$

9 $$SD\, = \,\sqrt {\sum\limits_{r = 1}^{N} {\left[ {\left( {y_{{_{2} }}^{\exp } - y_{{_{2} }}^{cal} } \right)_{r} \, - \,RE^{ave} } \right]^{2}} /N}$$

10 $$SR_{r} \, = \,RE_{r} /SD\,\,\,\,\,\,\,\,\,\,r = 1,\,2,\,...,N$$

11 $$H\, = \,\xi \,\left( {\xi^{T} \,\xi } \right)\,\xi^{ - 1} \quad \xi \,is\,the\,matrix\,of\,independent\,variables.$$

here, *RE*^*ave*^ and *SD* represent the average value of the residual error and standard deviation, respectively.

**Figure 9:**
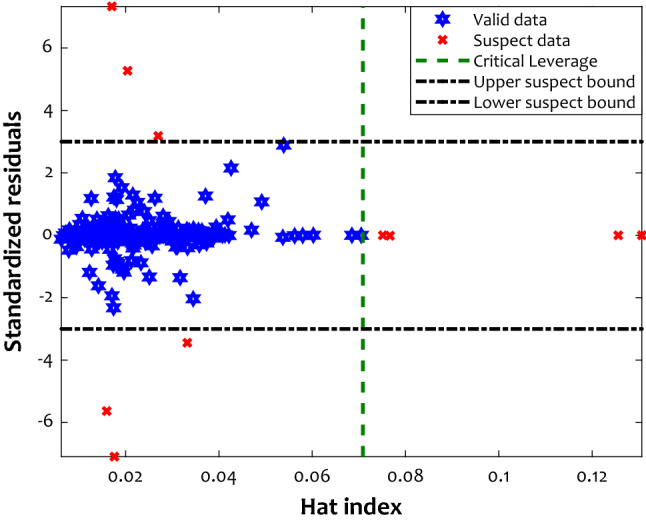
Analyzing the laboratory-measured solubility data for identifying valid and suspect information.

The consequence of applying the leverage tactic on the gathered database for anti-inflammatory drug-SCCO_2_ systems has been published in Fig. 9. Only one segment of Fig. 9 is valid, and all other five parts are suspect. This tactic confirms that 244 out of 254 experiments are valid, and the outlier may poison only less than four percent of the historical datasets. The accomplished analysis in this stage reveals that the collected databased used for model construction is mainly valid. Thus, the proposed ANFIS2-H is solely allowed to be used for estimating anti-inflammatory drug solubility in supercritical CO_2_ from molecular weight, melting temperature, pressure, solvent density, and temperature.

## Conclusion

This study systematically compared the prediction accuracy of 2150 intelligent estimators from three different categories (artificial neural networks, hybrid neuro-fuzzy, and support vector regression) to estimate anti-inflammatory drug solubility in supercritical CO_2_. The conducted comparisons approved that the adaptive neuro-fuzzy inference system with the subtractive clustering membership function (ANFIS2-H) has the highest accuracy for the considered objective. The cluster radius of this ANFIS2-H model adjusted by the hybrid optimization algorithm is 0.6111. The ANFIS2-H model estimated 254 laboratory-measured solubility data with the AAPRE = 3.13%, MSE = 2.58 × 10^–9^, and R^2^ = 0.99919. Furthermore, the AAPRE associated with each NSAID-SCCO_2_ phase equilibrium ranges from 1.04 to 6.05%. In addition, the LSSVR with the linear kernel function shows the worst predictive performance for estimating the NSAID’s solubility in the SCCO_2_. The relevancy analyses performed by three diverse scenarios justified that increasing the drug’s molecular weight and melting temperature decreases their solubility in supercritical CO_2_. In addition, experimental observations, modeling findings, and relevancy analyses indicated that increasing pressure, temperature, and SCCO_2_ density raise the drug solubility in supercritical solvents. The leverage methodology showed that only ten datasets are potential outliers, and all other experiments have been conducted on a valid basis. Both modeling and experimental observations clarified that the maximum and minimum tendency of the supercritical CO_2_ is devoted to the Ibuprofen and Naproxen drugs, respectively. Coupling the developed intelligent scenario with an optimization technique to precisely locate the operating conditions that maximize each anti-inflammatory drug’s solubility in supercritical carbon dioxide may be considered as a next research step in this field.

## References

[1] Karimi M, Vaferi B, Hosseini SH, Rasteh M (2018). Designing an efficient artificial intelligent approach for estimation of hydrodynamic characteristics of tapered fluidized bed from its design and operating parameters. Ind. Eng. Chem. Res..

[2] Marsousi S, Karimi-Sabet J, Moosavian MA, Amini Y (2019). Liquid–liquid extraction of calcium using ionic liquids in spiral microfluidics. Chem. Eng. J..

[3] Ghanbari Pakdehi S, Vaferi B (2016). A study on adsorptive removal of DMAZ from aqueous solutions by ZSM-5, NaY zeolites, and activated carbon. Desalin. Water Treat..

[4] Mahmoodi F, Darvishi P, Vaferi B (2018). Prediction of coefficients of the Langmuir adsorption isotherm using various artificial intelligence (AI) techniques. J. Iran. Chem. Soc..

[5] Amini Y, Gerdroodbary MB, Pishvaie MR, Moradi R, Monfared SM (2016). Optimal control of batch cooling crystallizers by using genetic algorithm. Case Stud. Therm. Eng..

[6] Rahimpour MR, Mazinani S, Vaferi B, Baktash MS (2011). Comparison of two different flow types on CO removal along a two-stage hydrogen permselective membrane reactor for methanol synthesis. Appl. Energy.

[7] Rahimpour MR, Baktash MS, Vaferi B, Mazinani S (2011). Reduction in CO emissions along a two-stage hydrogen-permselective membrane reactor in methanol synthesis process. J. Ind. Eng. Chem..

[8] Sadeghi A, Amini Y, Saidi MH, Yavari H (2015). Shear-rate-dependent rheology effects on mass transport and surface reactions in biomicrofluidic devices. AIChE J..

[9] Hassim N, Markom M, Rosli MI, Harun S (2021). Scale-up approach for supercritical fluid extraction with ethanol–water modified carbon dioxide on Phyllanthus niruri for safe enriched herbal extracts. Sci. Rep..

[10] Abusrafa AE, Challiwala MS, Choudhury HA, Wilhite BA, Elbashir NO (2020). Experimental verification of 2-dimensional computational fluid dynamics modeling of supercritical fluids Fischer Tropsch reactor bed. Catal. Today.

[11] Wang W (2021). Supercritical carbon dioxide applications in food processing. Food Eng. Rev..

[12] Meng Y, Su F, Chen Y (2016). Supercritical fluid synthesis and tribological applications of silver nanoparticle-decorated graphene in engine oil nanofluid. Sci. Rep..

[13] Tian X (2017). Shear-assisted production of few-layer boron nitride nanosheets by supercritical CO2 exfoliation and its use for thermally conductive epoxy composites. Sci. Rep..

[14] Liu P, Chen W, Liu C, Tian M, Liu P (2019). A novel poly (vinyl alcohol)/poly (ethylene glycol) scaffold for tissue engineering with a unique bimodal open-celled structure fabricated using supercritical fluid foaming. Sci. Rep..

[15] Pishnamazi M (2020). Using static method to measure tolmetin solubility at different pressures and temperatures in supercritical carbon dioxide. Sci. Rep..

[16] Fomin YD, Ryzhov VN, Tsiok EN, Brazhkin VV (2015). Dynamical crossover line in supercritical water. Sci. Rep..

[17] Xing F (2021). Accurate prediction of thermal conductivity of supercritical propane using LSSVM. Energy Sour. Part A Recover Util. Environ. Eff..

[18] Alaydi H, Downey P, McKeon-Bennett M, Beletskaya T (2021). Supercritical-CO 2 extraction, identification and quantification of polyprenol as a bioactive ingredient from Irish trees species. Sci. Rep..

[19] Lashkarbolooki M, Vaferi B, Shariati A, Zeinolabedini Hezave A (2013). Investigating vapor-liquid equilibria of binary mixtures containing supercritical or near-critical carbon dioxide and a cyclic compound using cascade neural network. Fluid Phase Equilib..

[20] Vaferi B, Lashkarbolooki M, Esmaeili H, Shariati A (2018). Toward artificial intelligence-based modeling of vapor liquid equilibria of carbon dioxide and refrigerant binary systems. J. Serb. Chem. Soc..

[21] Chen, L. *Handbook of Research on Advancements in Supercritical Fluids Applications for Sustainable Energy Systems* (IGI Global, 2020).

[22] Zabihi S (2020). Experimental solubility measurements of fenoprofen in supercritical carbon dioxide. J. Chem. Eng. Data.

[23] Coimbra P, Duarte CMM, De Sousa HC (2006). Cubic equation-of-state correlation of the solubility of some anti-inflammatory drugs in supercritical carbon dioxide. Fluid Phase Equilib..

[24] Suleiman D, Antonio Estévez L, Pulido JC, García JE, Mojica C (2005). Solubility of anti-inflammatory, anti-cancer, and anti-HIV drugs in supercritical carbon dioxide. J. Chem. Eng. Data.

[25] Macnaughton SJ (1996). Solubility of anti-inflammatory drugs in supercritical carbon dioxide. J. Chem. Eng. Data.

[26] Zabihi S, Esmaeili-Faraj SH, Borousan F, Hezave AZ, Shirazian S (2020). Loxoprofen solubility in supercritical carbon dioxide: experimental and modeling approaches. J. Chem. Eng. Data.

[27] Su C-S, Chen Y-P (2008). Measurement and correlation for the solid solubility of non-steroidal anti-inflammatory drugs (NSAIDs) in supercritical carbon dioxide. J. Supercrit. Fluids.

[28] Shojaee SA, Rajaei H, Hezave AZ, Lashkarbolooki M, Esmaeilzadeh F (2013). Experimental measurement and correlation for solubility of piroxicam (a non-steroidal anti-inflammatory drugs (NSAIDs)) in supercritical carbon dioxide. J. Supercrit. Fluids.

[29] Baghban A, Sasanipour J, Zhang Z (2018). A new chemical structure-based model to estimate solid compound solubility in supercritical CO2. J. CO2 Util..

[30] Hozhabr SB, Mazloumi SH, Sargolzaei J (2014). Correlation of solute solubility in supercritical carbon dioxide using a new empirical equation. Chem. Eng. Res. Des..

[31] Yang H, Zhong C (2005). Modeling of the solubility of aromatic compounds in supercritical carbon dioxide-cosolvent systems using SAFT equation of state. J. Supercrit. Fluids.

[32] Huang Z, Kawi S, Chiew YC (2004). Application of the perturbed Lennard-Jones chain equation of state to solute solubility in supercritical carbon dioxide. Fluid Phase Equilib..

[33] Sodeifian G, Saadati Ardestani N, Sajadian SA, Panah HS (2018). Measurement, correlation and thermodynamic modeling of the solubility of Ketotifen fumarate (KTF) in supercritical carbon dioxide. Fluid Phase Equilib..

[34] Sodeifian G, Razmimanesh F, Sajadian SA (2020). Prediction of solubility of sunitinib malate (an anti-cancer drug) in supercritical carbon dioxide (SC–CO2): Experimental correlations and thermodynamic modeling. J. Mol. Liq..

[35] Sodeifian G, Saadati Ardestani N, Sajadian SA, Golmohammadi MR, Fazlali A (2020). Prediction of solubility of sodium valproate in supercritical carbon dioxide: Experimental study and thermodynamic modeling. ACS Appl. Mater. Interfaces.

[36] Sodeifian G, Razmimanesh F, Saadati Ardestani N, Sajadian SA (2020). Experimental data and thermodynamic modeling of solubility of Azathioprine, as an immunosuppressive and anti-cancer drug, in supercritical carbon dioxide. J. Mol. Liq..

[37] Ramtin, A. R., Nain, P., Towsley, D., de Silva, E. S. & Menasche, D. S. Are covert ddos attacks facing multi-feature detectors feasible. *ACM SIGMETRICS Perform. Eval. Rev.* (2021).

[38] Jiang Y, Zhang G, Wang J, Vaferi B (2021). Hydrogen solubility in aromatic/cyclic compounds: Prediction by different machine learning techniques. Int. J. Hydrogen Energy.

[39] Karimi M, Vaferi B, Hosseini SH, Olazar M, Rashidi S (2020). Smart computing approach for design and scale-up of conical spouted beds with open-sided draft tubes. Particuology.

[40] Sanaat A, Zaidi H (2020). Depth of interaction estimation in a preclinical PET scanner equipped with monolithic crystals coupled to SiPMs using a deep neural network. Appl. Sci..

[41] Zou Y (2021). MK-FSVM-SVDD: a multiple kernel-based fuzzy SVM model for predicting DNA-binding proteins via support vector data description. Curr. Bioinform..

[42] Keshishian M (2020). Estimating and interpreting nonlinear receptive field of sensory neural responses with deep neural network models. Elife.

[43] Chenarlogh, V. A., Razzazi, F. & Mohammadyahya, N. A multi-view human action recognition system in limited data case using multi-stream CNN. In *2019 5th Iranian Conference on Signal Processing and Intelligent Systems (ICSPIS)* 1–11 (IEEE, 2019).

[44] Karimi, M., Jahanshahi, A., Mazloumi, A. & Sabzi, H. Z. Border gateway protocol anomaly detection using neural network. In *2019 IEEE International Conference on Big Data (Big Data)* 6092–6094 (IEEE, 2019).

[45] Li S (2021). Prediction of oral hepatotoxic dose of natural products derived from traditional Chinese medicines based on SVM classifier and PBPK modeling. Arch. Toxicol..

[46] Amini Y, Fattahi M, Khorasheh F, Sahebdelfar S (2013). Neural network modeling the effect of oxygenate additives on the performance of Pt–Sn/γ-Al 2 O 3 catalyst in propane dehydrogenation. Appl. Petrochem. Res..

[47] Ghanbari S, Vaferi B (2015). Experimental and theoretical investigation of water removal from DMAZ liquid fuel by an adsorption process. Acta Astronaut..

[48] Karimi M, Alibak AH, Alizadeh SMS, Sharif M, Vaferi B (2021). Intelligent modeling for considering the effect of bio-source type and appearance shape on the biomass heat capacity. Measurement.

[49] Vaferi B, Eslamloueyan R, Ayatollahi S (2015). Application of recurrent networks to classification of oil reservoir models in well-testing analysis. Energy Sour. Part A Recover Util. Environ. Eff..

[50] Moghimihanjani M, Vaferi B (2021). A combined wavelet transform and recurrent neural networks scheme for identification of hydrocarbon reservoir systems from well testing signals. J. Energy Resour. Technol..

[51] Cao Y, Kamrani E, Mirzaei S, Khandakar A, Vaferi B (2022). Electrical efficiency of the photovoltaic/thermal collectors cooled by nanofluids: Machine learning simulation and optimization by evolutionary algorithm. Energy Rep..

[52] Karimi M, Aminzadehsarikhanbeglou E, Vaferi B (2021). Robust intelligent topology for estimation of heat capacity of biochar pyrolysis residues. Measurement.

[53] Moosavi SR, Vaferi B, Wood DA (2021). Auto-characterization of naturally fractured reservoirs drilled by horizontal well using multi-output least squares support vector regression. Arab. J. Geosci..

[54] Zamani HA, Rafiee-Taghanaki S, Karimi M, Arabloo M, Dadashi A (2015). Implementing ANFIS for prediction of reservoir oil solution gas-oil ratio. J. Nat. Gas Sci. Eng..

[55] Isen E, Boran S (2018). A novel approach based on combining ANFIS, genetic algorithm and fuzzy c-means methods for multiple criteria inventory classification. Arab. J. Sci. Eng..

[56] Zhou Z, Davoudi E, Vaferi B (2021). Monitoring the effect of surface functionalization on the CO_2_ capture by graphene oxide/methyl diethanolamine nanofluids. J. Environ. Chem. Eng..

[57] Ghanbari S, Vaferi B (2017). Prediction of degree of crystallinity for the LTA zeolite using artificial neural networks. Mater. Sci. Pol..

[58] Ramtin AR, Nain P, Menasche DS, Towsley D, deSilva ES (2021). Fundamental scaling laws of covert DDoS attacks. Perform. Eval..

[59] Hosseini S, Vaferi B (2021). Determination of methanol loss due to vaporization in gas hydrate inhibition process using intelligent connectionist paradigms. Arab. J. Sci. Eng..

[60] Nabipour N, Qasem SN, Salwana E, Baghban A (2020). Evolving LSSVM and ELM models to predict solubility of non-hydrocarbon gases in aqueous electrolyte systems. Measurement.

